# Training Habits of Eumenorrheic Active Women during the Different Phases of Their Menstrual Cycle: A Descriptive Study

**DOI:** 10.3390/ijerph18073662

**Published:** 2021-04-01

**Authors:** Felipe García-Pinillos, Pascual Bujalance-Moreno, Daniel Jérez-Mayorga, Álvaro Velarde-Sotres, Vanessa Anaya-Moix, Silvia Pueyo-Villa, Carlos Lago-Fuentes

**Affiliations:** 1 Department of Physical Education, Sports and Recreation, Universidad de La Frontera, Temuco 4811230, Chile; fgpinillos@ugr.es; 2 Faculty of Sports Sciences, University of Granada, 18010 Granada, Spain; 3 Department of Corporal Expression, University of Jaen, 23071 Jaen, Spain; pascualbujalancemoreno@gmail.com; 4 Facultad de Ciencias de la Rehabilitación, Universidad Andrés Bello, Santiago 7591538, Chile; daniel.jerez@unab.cl; 5 Faculty of Health Sciences, Universidad Europea del Atlántico, 39011 Santander, Spain; alvaro.velarde@uneatlantico.es; 6 Department of Education, Universidad Internacional Iberoamericana, Campeche 24560, Mexico; vanessa.anaya@uneatlantico.es (V.A.-M.); silvia.pueyo@uneatlantico.es (S.P.-V.); 7 Department of Languages and Education, Universidad Europea del Atlántico, 39011 Santander, Spain

**Keywords:** gender, training load, health surveys, sport participation

## Abstract

The purpose of this study was to examine the training habits of eumenorrheic active women during their menstrual cycle (MC), and its perceived influence on physical performance regarding their athletic level. A group of 1250 sportswomen filled in a questionnaire referring to demographic information, athletic performance and MC-related training habits. Of the participants, 81% reported having a stable duration of MC, with most of them (57%) lasting 26–30 days. Concerning MC-related training habits, 79% indicated that their MC affects athletic performance, although 71% did not consider their MC in their training program, with no differences or modifications in training volume or in training intensity for low-level athletes (LLA) and high-level athletes (HLA) with hormonal contraceptive (HC) use. However, LLA with a normal MC adapted their training habits more, compared with HLA, also stopping their training (47.1% vs. 16.1%, respectively). Thus, different training strategies should be designed for HLA and LLA with a normal MC, but this is not so necessary for HLA and LLA who use HC. To sum up, training adaptations should be individually designed according to the training level and use or non-use of HC, always taking into account the pain suffered during the menstrual phase in most of the athletes.

## 1. Introduction

The presence and popularity of physical activity and sport for women has considerably increased in the last few decades, and therefore, the need to improve knowledge about their physiology and adaptations to exercise has become crucial. Traditionally, the physiological responses to exercise were assumed to be equal between the sexes [1], and so the recommendations about sport practice and prescription for women have been generalized for decades without even testing whether these guidelines were correct. A potential rationale for this underrepresentation is the complexities and methodological difficulties related to the menstrual cycle (MC) [1]. Based on that argument, sports sciences have been focused on men, or have included women without considering the MC’s influence [2].

A typical MC lasts around 28 days and consists of a follicular phase (i.e., characterized by 12–14 days’ duration, high levels of estrogen and low progesterone), ovulation phase (i.e., 1–3 days’ duration and preceded by a second increase in estrogen), and a luteal phase (i.e., 12–14 days’ duration, with high levels of progesterone and medium levels of estrogen) [3]. Plentiful information has been made available about the role of estrogen and progesterone in the female physiology during the last few decades. Regarding estrogen, it has been demonstrated that it increases the muscle glycogen storage capacity as well as increasing free fatty acid availability, and it is used as a fuel source through the use of oxidative pathways [4]. From a physiological standpoint, this is very important because it leads to decreased carbohydrate use, or glycogen sparing [5], and it therefore decreases the dependence on anaerobic pathways for Adenosine triphosphate (ATP) production. In practical terms, high estrogen levels are associated with lower blood lactate levels and longer times to exhaustion [5]. Progesterone is known to have a sympathetic effect (e.g., it increases resting heart rate [6], basal body temperature and ventilation) [7]. This effect plays a key role during exercise because it seems to increase the perceived exertion and decrease athletic performance, especially in hot and/or humid environments [8]. Beyond these physiological effects, what is crucial to understand about progesterone is its ability to antagonize estrogenic effects [1,9]. In this regard, a previous study showed that high progesterone levels can inhibit the enhancement of the carbohydrate metabolism promoted by estradiol, which is a primary estrogen [10].

Taking into consideration the above information, it seems clear that both hormones have different target organs and create diverse biochemical and physiological environments, which have been suggested to be determinant in exercise capacity and thus adaptations to training [8,11,12]. Nevertheless, there is no consensus about the influence of hormone variations induced by the MC’s phases on physical performance, with other works reporting no effect [13,14].

Once the physiological findings are revealed, the next question is to learn the influence of these changes in athletes. For this, the use of questionnaires to analyze vast populations is a common strategy in sports science. In this regard, some previous studies have used self-reported questionnaires to examine topics related to the MC [15,16]. Martin et al. [16] aimed to determine the prevalence of hormonal contraceptive (HC) use and the side effects experienced by users and non-users in elite female athletes, whereas Bruinvels et al. [15] focused on heavy menstrual bleeding (HMB) and its perceived effects on training and performance. However, to the best of the authors’ knowledge, no previous studies have examined the influence of their MC and their phases on training habits in eumenorrheic active women.

Since female athletes keep training and competing whilst having to manage hormone alterations and their effects, a description of what female athletes are doing and how they manage the potential effects of the MC might be of interest for coaches, athletes and sports scientists. Although research based on a questionnaire can show some limitations, this is the best option to make a survey with a large sample size, with information directly collected by sportswomen regarding their own MC and its influence on their training habits. For these reasons, the purpose of this study is to examine the training habits of eumenorrheic active women during their MC and its perceived influence on physical performance. The current study also aims to investigate the differences in MC-related training habits regarding the athletic level and the use or non-use of hormonal contraceptives (HC).

## 2. Materials and Methods

### 2.1. Subjects

One thousand, two hundred and fifty eumenorrheic active women voluntarily participated in this study (age range: 18-45 years; age mean: 27.8 ± 6.6 years). All participants met the inclusion criteria: (1) older than 18 and younger than 45 years old, and (2) with three or more training sessions per week [17]. This study meets the ethical standards of the World Medical Association’s Declaration of Helsinki (2013), and it was approved by the Institutional Review Board (Universidad de La Frontera, Temucho, Chile, 005_19).

### 2.2. Procedure

A cross-sectional study with a descriptive purpose was performed. An ad-hoc questionnaire was designed for a massive mailshot to physically active women through an online Google Form (https://drive.google.com/open?id=1Pw5AecF3Wqhn-1dn13IhqZxZW2QjiyxTtkmsz55Bs28) (accessed on 15 September 2019). This research project was conducted according to the European General Data Protection Regulation (2018).

After receiving ethical approval from the Institutional Review Board, pilot tests were conducted with a small sample of participants (*n* = 40) to evaluate the clarity and content of the online Google Forms. All participants involved in the pilot test indicated that the questionnaire was appropriate and suitable. Subsequently, sports centers, sports clubs, federations and sports institutes in Spain were contacted through their administrators and asked to publicize the study to their athletes and clients as long as those sports organizations were in line with the current data protection regulations, implying that athletes were informed about the potential use of their personal data for research purposes when they provided such information. Then, athletes who were willing to participate in the study were given a link to the online questionnaire.

According to online informed consent procedures, participants were told of the purpose and details of the study through a participant information sheet. Participants were informed that all responses would be kept strictly confidential and would only be used for the purposes of the study. Having consented to participation in the study, participants filled in seventeen items split into four sections: (i) demographic information (i.e., age), (ii) information about athletic performance in the last 6 months (i.e., to have a coach, to be federated, sport modality and athletic level), (iii) information about their training habits in the last 6 months (i.e., hours and sessions per week), and (iv) information about their MC and MC-related training habits (i.e., age at menarche, duration and regularity of their MC, use of HC, the perceived influence of their MC on physical performance, MC-related modifications in training plans, and pain influence).

### 2.3. Statistical Analysis

Descriptive data are presented as means and standard deviation for interval variables, and as frequency and percentage for nominal variables. Six level groups were determined according to self-reported athletic level (non-competitive level or level 0, local or level 1, autonomic or level 2, national or level 3, international or level 4, and elite or level 5). To compare differences in the adaptation of the parameters of training during the MC, the six groups were dichotomized into two according to their athletic level: lower-level athletes (LLA) (groups 0, 1 and 2), and higher-level athletes (HLA) (groups 3, 4 and 5). The chi-squared test was conducted to determine differences between level groups with and without HC. All statistical analyses were performed using the software package SPSS (IBM SPSS version 22.0, Chicago, IL, USA). The effect size was calculated following previous studies [18].

## 3. Results

As general information about this group of 1250 women, the results obtained indicate that participants trained 6.7 ± 3.4 h per week, distributed over 4.3 ± 2.1 sessions per week (Table 1). Among these women, 64.7% had a coach and 33.6% were federated. The most practiced sport modalities were team sports (25.9%), athletics (24.6%) and fitness-related activities (19.7%), whereas other modalities such as CrossFit (9.8%), cycling and dancing (5.8%) also showed moderate levels of practice among active women. The self-reported age at menarche was 12.7 ± 1.7 years.

Concerning the athletic level, regardless of sport modality, most of the surveyed women (60.7%) indicated a non-competitive level (level 0), whereas the rest of the participants reported a competitive level (at local level or level 1–9.8%; autonomic or level 2–7.7%; national or level 3–18.7%; international or level 4–2.4%; and elite or level 5–0.6%).

Regarding the profile of the MC in relation to the different athletic levels (Table 2), 81% reported having a stable duration of MC with most of them (57%) lasting 26–30 days. No statistical differences were found in the length of the MC when comparing different athletic levels, except with the elite level (*n* = 8, *p* < 0.001, effect size (ES) = 0.42). Regarding the regularity of the MC, international athletes reported a lower percentage (60% regularity) compared with the rest of the groups. In general, 28% reported using an HC method, showing that 43.8% of the level 2 group used HC.

Table 3 includes a comparison between different athletic levels (HLA vs. LLA) with a normal cycle (no HC use). Of the LLA, 80.3% indicated that their MC affects athletic performance, with statistical differences compared to HLA. However, almost 70% did not consider the MC in their training program for both groups. Regarding training volume and intensity, LLA affected both variables more during their menstrual phase (MP) compared with HLA (*p* < 0.005 and *p* < 0.001, respectively). Almost 50% of the LLA stopped their training during their MP, while <20% of HLA did so (*p* < 0.001; ES = 0.84). Lastly, among the sportswomen, 55% to 63% reported that they suffered pain during their MP, without differences between both groups.

Table 4 describes the influence of the MC on training habits, comparing HLA and LLA athletes. Both LLA and HLA athletes consuming HC reported their performance being affected. However, only 9.1% of HLA reported no effect, against 28% of LLA (*p* < 0.005; ES = 0.47). Regarding the rest of the variables, no statistical differences were reported when comparing both groups, considering the effect of the MC in their training (*p* = 0.669), neither adapting their volume or training, nor stopping training during their MP (*p* = 0.656, *p* = 0.09 and *p* = 0.143, respectively). Finally, both groups suffered pain during their MP, with between 55 and 63% of athletes reporting it, with no differences between groups.

## 4. Discussion

This study aimed to examine the training habits of eumenorrheic active women during their MC and its perceived influence on their physical performance. The results obtained indicate that, despite a high percentage of the surveyed women confirming that the MC affects physical performance and reporting feeling pain during the MP, most of them reported making no changes to their training programs during the MC, with no modifications in training volume or intensity during the MP. This information is of interest as it reinforces the importance of the MC in the training plans of eumenorrheic active women.

Until now, there has been no clear evidence about the relationship between MC and sports performance in different physical outcomes [13,16,19,20,21]. Regarding this, almost 80% of athletes indicated that the MC affects their performance, with more influence on LLA with a normal MC and HLA with HC consumption. Athletes also suffered pain during the MP, with no differences between groups, independent of the use or non-use of HC. These results match previous studies that indicated most athletes suffered negative side effects during the early days of the MP, affecting their sports performance throughout the cycle [15,16] because of HMB, stomach cramps, back pain and headaches, among other symptoms. Conversely, exercise has been shown to improve the symptoms of premenstrual syndrome, inter alia [22], so female athletes should consider managing training variables (e.g., volume and intensity) during the MC, especially during the MP, but not stop training.

Given the lack of consensus about the influence of the hormone variations associated with the MC, the results provided by the current work might be of interest for coaches and athletes. For instance, whereas it has been suggested that age at menarche might be influenced by the level of sports practice and specialization in the sport [23], the data provided by this current study indicate that age at menarche was similar for all the sports in the study. About this, a previous study suggested that, in gymnastics, menarcheal age was delayed compared with other athletes [23]. However, the authors could not justify this finding regarding training levels, so according to our results, menarcheal age could not be influenced by training levels [23]. Further longitudinal studies should be performed with young female athletes to analyze possible changes to the menarcheal age and the influence of training levels. In this context, another interesting finding is the lack of differences in the length of the MC between level groups. Martin et al. [16] showed that the length of the MC can vary regarding athletic level, with some negative side effects such as primary dysmenorrhea. However, this does not match our results, with more than 50% of HLA reporting a duration of 26 to 30 days. Furthermore, most groups reported a high regularity of the MC, which is key to female performance and the first step for designing training programs according to their different phases [19]. Lastly, only 27.5% used HCs, which is a low prevalence compared with previous studies, and almost 60% of the British elite athletes [16]. HC can influence the regularity of the MC as well as reducing dysmenorrhea, but it also affects sport performance [16].

Additionally, this study also aimed to investigate the differences in MC-related training habits concerning athletic level, comparing results with and without the use of HC. Regarding this, it has been suggested that athletic level can influence the MC [16]. Firstly, regarding athletes with a natural MC (no HC use), the HLA adapted their training much less according to their MC than did the LLA (i.e., the LLA skipped more training sessions than HLA). Intensity, one of the main factors to control during the MC [20], was modified in 48.6% of the LLA compared with only 30.8% in the HLA group during the MP. That is, the HLA did not modify their workouts, regardless of the negative side effects (such as pain) associated with the MP during their preparation. Okano et al. [24] showed that the athletic level influenced the prevalence of eating disorders in Japanese and Chinese athletes, revealing that HLA are more prone to suffering them. So, training at a high level without considering hormone fluctuations during the MC (and especially the negative side effects before and during the MP) could be dangerous for female health and also increase injury risk [25]. In fact, a recent study stated that periodizing the strength training according to the different phases of the MC improves the gaining of lean body mass compared with traditional training [26]. Secondly, comparing both athletic levels via HC use reported different findings. In general, both groups felt pain during the MP, without statistical differences. No differences were also reported regarding the adaptation of training habits in either variable (intensity and volume) during the MP. Curiously, 90% of HLA with HC reported an effect on their performance, compared with only 72% of LLA. In this sense, a recent systematic review suggested that performance did not differ between the different phases of the MC in HC athletes, but their performance can be slightly inferior compared with a non-HC user [27]. Taking into account that HLA athletes using HC felt more effect on their performance compared with HLA with a natural MC, and the same percentage of pain was reported, HC might not be the best option for top athletes, due to the negative side effects of its use [27].

In summary, the results indicate that, despite a high percentage of the surveyed women confirming that the MC affects physical performance and feeling pain during their MP, most of them reported making no changes to their training programs during the MC, with no modifications in training volume nor intensity during the MP. Comparing HC use to non-use, LLA with a natural MC adapted their training variables more during the MP compared to HLA. On the other hand, athletes using HC did not differ in their training adaptations regarding their athletic level. Apart from these key points, it is relevant to take into account that more than 50% of athletes (in both groups) suffered pain during the MP. This information is essential for coaches and practitioners to understand and adapt training loads when pain is present during the MC. Further strategies should analyze different adaptations of training plans throughout the MC, and particularly during the MP, to as far as possible reduce the pain and optimize their sport performance. For these reasons, monitoring and programming training loads according to athletic level, their type of MC (use or non-use of HC), and the different phases of the MC might be relevant during the training process according to these results. Future studies should apply different intensities and frequencies during the MP to compare the effects on sport performance, especially in a natural MC.

## 5. Conclusions

The novel aspect of this research regards the level of influence of the menstrual cycle on the performance and training habits of eumenorrheic active women. Defining and knowing the side effects during each phase of the menstrual cycle at different performance levels and sports modalities should be relevant to adapting training programs properly and reducing non-practice times during the menstruation phase, both in HC and non-HC users. For this reason, staff and physical education coaches should be aware of the importance of managing and registering the menstrual phases of women to optimize their training and adapt training loads, especially during the bleeding or menstrual phase. Moreover, these results could also be useful to compare different performances regarding sports modalities.

To sum up, this study provides descriptive information about the MC of eumenorrheic active women, and the modifications performed in their training programs in relation to the different phases of the MC. Given the reported hormonal changes during the different phases of the MC, both sports scientists and coaches must pay special attention to the role of this factor, and also to their negative side effects in some phases, suggesting the need for an educational process and a constant dialog with their athletes about their feelings, negative side effects (if they exist) and pain during the MP, independently of the use or non-use of HC.

## Figures and Tables

**Table 1:** Descriptive data of active women related to sport modality.

	N	Age	Sessions/Week	Hours/Week	Age of Menarche
Athletic	308	29.8 ± 0.4	4.7 ± 0.1	6.7 ± 0.2	13.1 ± 0.1
CrossFit	123	28.4 ± 0.7	4.6 ± 0.3	6.1 ± 0.3	12.6 ± 0.2
Cycling	72	32.4 ± 0.8	4.2 ± 0.2	7.6 ± 0.4	12.4 ± 0.2
Dancing	72	29.5 ± 0.7	5.0 ± 0.5	6.0 ± 0.4	12.2 ± 0.2
Equestrian	6	25.0 ± 1.8	4.5 ± 0.7	8.5 ± 1.6	12.5 ± 0.2
Fight sports	15	26.2 ± 0.7	3.0 ± 0.2	6.0 ± 0.7	12.0 ± 0.3
Fitness	246	27.9 ± 0.4	3.7 ± 0.7	5.6 ± 0.2	12.6 ± 0.1
Gymnastics	18	26.2 ± 0.9	3.0 ± 0.3	5.0 ± 0.9	12.7 ± 0.3
Racquet sports	36	25.2 ± 0.8	3.6 ± 0.2	4.8 ± 0.3	13.0 ± 0.3
Swimming	15	27.4 ± 0.8	4.6 ± 0.2	7.8 ± 0.8	12.6 ± 0.4
Team sports	324	24.5 ± 0.3	4.2 ± 0.9	8.2 ± 0.2	12.7 ± 0.1
Winter sports	9	28.7 ± 1.2	3.0 ± 0.2	5.3 ± 0.3	13.0 ± 0.8

**Table 2:** Profile of the menstrual cycle (MC) related to different athletic levels.

Variables		LG0 (*n* = 759)	LG1 (*n* = 123)	LG2 (*n* = 96)	LG3 (*n* = 234)	LG4 (*n* = 30)	LG5 (*n* = 8)	*p*-Value	ES
Length of MC	21–25 days	153 (20.2)	30 (24.4)	24 (25.0)	51 (21.8)	6 (20.0)	0 (0.0)	<0.001	0.42
	26–30 days	453 (59.7)	54 (43.9)	54 (56.2)	129 (55.1)	9 (30.0)	8 (100.0)		
	31–35 days	81 (10.7)	27 (22.0)	6 (6.2)	18 (7.7)	6 (20.0)	0 (0.0)		
	It varies a lot	72 (9.5)	12 (9.8)	12 (12.5)	36 (15.4)	9 (30.0)	0 (0.0)		
Regularity of MC	Yes	633 (83.4)	99 (80.5)	72 (75.0)	189 (80.8)	18 (60.0)	8 (100.0)	0.008	0.10
	No	126 (16.6)	24 (19.5)	24 (25.0)	45 (19.2)	12 (40.0)	0 (0.0)		
HC	Yes	204 (26.9)	33 (26.8)	42 (43.8)	60 (25.6)	6 (20.0)	0 (0.0)	<0.001	0.26
	No	553 (72.9)	90 (73.2)	54 (56.2)	174 (74.4)	22 (73.3)	8 (100.0)		

Note: percentages are calculated according to the number of sportswomen per level group. ES: effect size, MC: menstrual cycle; HC: hormonal contraceptives; LG0: non-competitive level; LG1: local level; LG2: autonomic level; LG3: national level; LG4: international level; LG5: elite level.

**Table 3:** Influence of the menstrual cycle (MC) on training habits, related to different athletic levels without contraceptive hormones (*n* = 905).

Variables		LLA (*n* = 697)	HLA (*n* = 208)	*p*-Value	ES
Effect on performance	No	138 (19.8)	48 (23.1)	<0.05	0.17
	Yes—moderately	397 (57.0)	128 (61.5)		
	Yes—strongly	162 (23.2)	32 (15.4)		
Considering MC in training	Yes	210 (30.1)	64 (30.8)	0.86	0.01
	No	487 (69.7)	144 (69.2)		
Adapting volume during MP	Yes	264 (37.9)	55 (26.4)	<0.005	0.29
	No	433 (62.1)	153 (73.6)		
Adapting intensity during MP	Yes	339 (48.6)	64 (30.8)	<0.001	0.42
	No	358 (51.4)	144 (69.2)		
Stopping training during MP	Yes	330 (47.3)	34 (16.3)	<0.001	0.84
	No	367 (52.7)	174 (83.7)		
Pain during MP	Yes	459 (65.9)	128 (61.5)	0.256	0.10
	No	238 (34.1)	80 (38.5)		

Note: percentages are calculated related to the number of sportswomen per level group. ES: effect size; MP: menstrual phase; MC: menstrual cycle; HLA: higher-level athletes, LLA: lower-level athletes.

**Table 4:** Influence of the menstrual cycle (MC) on training habits, related to different athletic levels with contraceptive hormones (*n* = 345).

Variables		LLA (*n* = 279)	HLA (*n* = 66)	*p*-Value	ES
Effect on performance	No	78 (28.0)	6 (9.1)	<0.005	0.47
	Yes—moderately	147 (52.7)	39 (59.1)		
	Yes—strongly	54 (19.3)	21 (31.8)		
Considering MC in training	Yes	69 (24.7)	18 (27.3)	0.669	0.06
	No	210 (75.3)	48 (72.7)		
Adapting volume during MP	Yes	81 (29.0)	21 (31.8)	0.656	0.06
	No	198 (71.0)	45 (68.2)		
Adapting intensity during MP	Yes	120 (43.0)	21 (31.8)	0.09	0.22
	No	159 (57.0)	45 (68.2)		
Stopping training during MP	Yes	75 (26.9)	12 (18.2)	0.143	0.20
	No	204 (73.1)	54 (81.8)		
Pain during MP	Yes	156 (55.9)	42 (63.6)	0.254	0.16
	No	123 (44.1)	24 (36.4)		

Note: percentages are calculated related to the number of sportswomen per level group. ES: effect size; MP: menstrual phase; MC: menstrual cycle; HLA: higher-level athletes, LLA: lower-level athletes.

## Data Availability

Not applicable.
